# 3-(2-Hydroxy­benzyl­ideneamino)benzonitrile

**DOI:** 10.1107/S1600536808005242

**Published:** 2008-02-29

**Authors:** Hai-Jun Xu, Xing-Xuan Gong, Han Wang

**Affiliations:** a Ordered Matter Science Research Center, College of Chemistry and Chemical Engineering, Southeast University, Nanjing 210096, People’s Republic of China

## Abstract

In the title mol­ecule, C_14_H_10_N_2_O, an intra­molecular O—H⋯N hydrogen bond contributes to the essential coplanarity of the two benzene rings, which form a dihedral angle of 6.04 (18)°.

## Related literature

For related crystal structures, see: Kosar *et al.* (2005 ▶); Cheng *et al.* (2005 ▶, 2006 ▶).
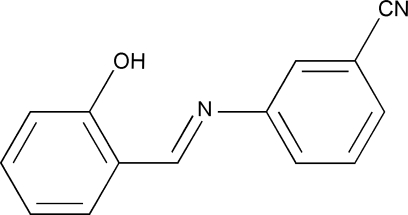


## Experimental

### 

#### Crystal data

C_14_H_10_N_2_O
*M* *_r_* = 222.24
Orthorhombic, 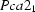

*a* = 26.397 (5) Å
*b* = 3.9211 (8) Å
*c* = 10.773 (2) Å
*V* = 1115.1 (4) Å^3^
*Z* = 4
Mo *K*α radiation
μ = 0.09 mm^−1^
*T* = 293 (2) K
0.22 × 0.05 × 0.05 mm

#### Data collection

Rigaku Mercury2 diffractometer
Absorption correction: multi-scan (*CrystalClear*; Rigaku, 2005 ▶) *T*_min_ = 0.812, *T*_max_ = 1.000 (expected range = 0.809–0.996)
9995 measured reflections
1339 independent reflections
901 reflections with *I* > 2σ(*I*)
*R*_int_ = 0.116

#### Refinement

*R*[*F* ^2^ > 2σ(*F* ^2^)] = 0.056
*wR*(*F* ^2^) = 0.121
*S* = 1.05
1339 reflections
160 parameters
2 restraints
H atoms treated by a mixture of independent and constrained refinement
Δρ_max_ = 0.15 e Å^−3^
Δρ_min_ = −0.16 e Å^−3^

### 

Data collection: *CrystalClear* (Rigaku, 2005 ▶); cell refinement: *CrystalClear*; data reduction: *CrystalClear*; program(s) used to solve structure: *SHELXS97* (Sheldrick, 2008 ▶); program(s) used to refine structure: *SHELXL97* (Sheldrick, 2008 ▶); molecular graphics: *SHELXTL* (Sheldrick, 2008 ▶); software used to prepare material for publication: *SHELXTL*.

## Supplementary Material

Crystal structure: contains datablocks I, global. DOI: 10.1107/S1600536808005242/cv2384sup1.cif

Structure factors: contains datablocks I. DOI: 10.1107/S1600536808005242/cv2384Isup2.hkl

Additional supplementary materials:  crystallographic information; 3D view; checkCIF report

## Figures and Tables

**Table 1:** Hydrogen-bond geometry (Å, °)

*D*—H⋯*A*	*D*—H	H⋯*A*	*D*⋯*A*	*D*—H⋯*A*
O1—H1*B*⋯N1	0.82 (2)	1.89 (4)	2.623 (4)	149 (7)

**Fig. 1.**
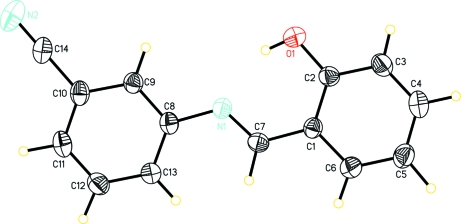
The molecular structure of the title compound with atomic numbering and displacement ellipsoids drawn at the 30% probability level.

## supplementary crystallographic information

### Comment

Schiff base compounds have attracted great attention for many years. These compounds play an important role in the development of coordination chemistry related to catalysis and enzymatic reactions, magnetism, photochromism and thermochromism. Here, we report the crystal structure of the title compound.

In the title compound (Fig. 1), all bond lengths are within normal ranges. The C7=N1 bond length of 1.277 (4)Å is a typical double bond, similar to the corresponding bond lengths in 4-methoxy-2-[(4-nitrophenyl)iminomethyl]phenol (Kosar *et al.*, 2005). The molecule is almost planar and displays a *trans* configuration with respect to the C7=N1 double bond. The dihedral angle between the benzene rings is 6.04 (18)°. Strong intramolecular O—H···N hydrogen-bond interaction (Talbe I), similar to the reported earlier (Cheng *et al.*, 2005, 2006), is observed in the molecule.

### Experimental

3-Aminobenzonitrile and salicylaldehyde were available commercially and were used without further purification. 3-Aminobenzonitrile (1.18 g, 10 mmol) and salicylaldehyde (1.22 g, 10 mmol) were dissolved in ethanol (20 ml). The mixture was heated to reflux for 4 h, then cooled to room temperature overnight and large amounts of a yellow precipitate were formed. Yellow crystals were obtained by recrystallization from ethyl alcohol (yield: 82%). For the X-ray diffraction analysis, suitable single crystals were obtained after one week by slow evaporation from an ethyl alcohol solution.

### Refinement

C-bound H atoms were geometrically positioned (C—H 0.93 Å) and refined as riding with *U*_iso_(H)= 1.2Ueq(C). Atom H1B was located on a difference map and refined isotropically with bon restraint O1—H1B = 0.82 (2) Å. In the absence of significant anomalous scatterers, 1124 Friedel pairs were merged.

### Crystal data

**Table:**

C_14_H_10_N_2_O	*F*_000_ = 464
*M**_r_* = 222.24	*D*_x_ = 1.324 Mg m^−^^3^
Orthorhombic, *P**c**a*2_1_	Mo *K*α radiation λ = 0.71073 Å
Hall symbol: P 2c -2ac	Cell parameters from 8294 reflections
*a* = 26.397 (5) Å	θ = 3.0–27.6º
*b* = 3.9211 (8) Å	µ = 0.09 mm^−^^1^
*c* = 10.773 (2) Å	*T* = 293 (2) K
*V* = 1115.1 (4) Å^3^	Stick, yellow
*Z* = 4	0.22 × 0.05 × 0.05 mm

### Data collection

**Table:**

Rigaku Mercury2 diffractometer	1339 independent reflections
Radiation source: fine-focus sealed tube	901 reflections with *I* > 2σ(*I*)
Monochromator: graphite	*R*_int_ = 0.116
Detector resolution: 13.6612 pixels mm^-1^	θ_max_ = 27.5º
*T* = 293(2) K	θ_min_ = 3.1º
ω scans	*h* = −34→34
Absorption correction: multi-scan(CrystalClear; Rigaku, 2005)	*k* = −5→5
*T*_min_ = 0.812, *T*_max_ = 1.00	*l* = −13→13
9995 measured reflections	

### Refinement

**Table:**

Refinement on *F*^2^	Secondary atom site location: difference Fourier map
Least-squares matrix: full	Hydrogen site location: inferred from neighbouring sites
*R*[*F*^2^ > 2σ(*F*^2^)] = 0.056	H atoms treated by a mixture of independent and constrained refinement
*wR*(*F*^2^) = 0.121	*w* = 1/[σ^2^(*F*_o_^2^) + (0.0413*P*)^2^] where *P* = (*F*_o_^2^ + 2*F*_c_^2^)/3
*S* = 1.05	(Δ/σ)_max_ < 0.001
1339 reflections	Δρ_max_ = 0.15 e Å^−^^3^
160 parameters	Δρ_min_ = −0.16 e Å^−^^3^
2 restraints	Extinction correction: none
Primary atom site location: structure-invariant direct methods	

### Special details

**Table:**

Geometry. All e.s.d.'s (except the e.s.d. in the dihedral angle between two l.s. planes) are estimated using the full covariance matrix. The cell e.s.d.'s are taken into account individually in the estimation of e.s.d.'s in distances, angles and torsion angles; correlations between e.s.d.'s in cell parameters are only used when they are defined by crystal symmetry. An approximate (isotropic) treatment of cell e.s.d.'s is used for estimating e.s.d.'s involving l.s. planes.
Refinement. Refinement of *F*^2^ against ALL reflections. The weighted *R*-factor *wR* and goodness of fit *S* are based on *F*^2^, conventional *R*-factors *R* are based on *F*, with *F* set to zero for negative *F*^2^. The threshold expression of *F*^2^ > σ(*F*^2^) is used only for calculating *R*-factors(gt) *etc*. and is not relevant to the choice of reflections for refinement. *R*-factors based on *F*^2^ are statistically about twice as large as those based on *F*, and *R*- factors based on ALL data will be even larger.

### Fractional atomic coordinates and isotropic or equivalent isotropic displacement parameters (Å^2^)

**Table:**

	*x*	*y*	*z*	*U*_iso_*/*U*_eq_	
N1	0.45687 (11)	0.2218 (7)	0.2697 (3)	0.0473 (8)	
C1	0.54425 (12)	0.2407 (8)	0.3273 (3)	0.0427 (8)	
C9	0.37076 (12)	0.2616 (9)	0.2071 (3)	0.0457 (9)	
H9A	0.3823	0.3718	0.1360	0.055*	
C6	0.57995 (14)	0.1614 (9)	0.4188 (3)	0.0559 (10)	
H6A	0.5694	0.0602	0.4924	0.067*	
C8	0.40534 (12)	0.1480 (8)	0.2952 (3)	0.0451 (8)	
C7	0.49124 (12)	0.1608 (8)	0.3501 (3)	0.0460 (9)	
H7A	0.4821	0.0624	0.4254	0.055*	
C3	0.61133 (14)	0.4649 (10)	0.2017 (3)	0.0570 (11)	
H3A	0.6223	0.5715	0.1294	0.068*	
C2	0.56072 (13)	0.3894 (9)	0.2167 (3)	0.0468 (9)	
C12	0.33597 (14)	−0.0682 (10)	0.4162 (4)	0.0601 (10)	
H12A	0.3245	−0.1800	0.4869	0.072*	
C10	0.31905 (13)	0.2118 (9)	0.2243 (3)	0.0510 (10)	
C11	0.30193 (13)	0.0476 (9)	0.3297 (3)	0.0572 (11)	
H11A	0.2674	0.0157	0.3421	0.069*	
C14	0.28378 (13)	0.3427 (10)	0.1343 (4)	0.0585 (10)	
O1	0.52789 (11)	0.4688 (8)	0.1250 (3)	0.0683 (8)	
C5	0.63050 (14)	0.2312 (10)	0.4013 (4)	0.0629 (11)	
H5A	0.6540	0.1756	0.4622	0.076*	
C4	0.64585 (14)	0.3839 (10)	0.2928 (4)	0.0632 (11)	
H4A	0.6800	0.4331	0.2808	0.076*	
C13	0.38733 (13)	−0.0193 (10)	0.3986 (3)	0.0542 (10)	
H13A	0.4101	−0.1006	0.4575	0.065*	
N2	0.25591 (14)	0.4542 (10)	0.0648 (4)	0.0896 (13)	
H1B	0.4999 (10)	0.396 (12)	0.145 (7)	0.13 (2)*	

### Atomic displacement parameters (Å^2^)

**Table:**

	*U*^11^	*U*^22^	*U*^33^	*U*^12^	*U*^13^	*U*^23^
N1	0.0426 (18)	0.0517 (18)	0.0474 (16)	0.0006 (13)	−0.0038 (14)	−0.0007 (13)
C1	0.038 (2)	0.0449 (17)	0.045 (2)	0.0021 (14)	0.0015 (17)	−0.0035 (16)
C9	0.042 (2)	0.048 (2)	0.046 (2)	0.0029 (16)	0.0022 (17)	−0.0027 (17)
C6	0.053 (2)	0.058 (2)	0.057 (2)	0.0006 (18)	−0.005 (2)	0.0031 (19)
C8	0.0392 (19)	0.0456 (19)	0.051 (2)	−0.0018 (15)	0.0032 (17)	−0.0088 (17)
C7	0.052 (2)	0.0445 (19)	0.042 (2)	0.0000 (16)	0.0045 (17)	0.0015 (17)
C3	0.054 (3)	0.061 (3)	0.056 (2)	−0.0065 (18)	0.0091 (19)	−0.0002 (19)
C2	0.047 (2)	0.051 (2)	0.042 (2)	−0.0009 (16)	0.0012 (16)	−0.0012 (18)
C12	0.056 (2)	0.065 (3)	0.060 (2)	−0.0083 (19)	0.006 (2)	0.006 (2)
C10	0.042 (2)	0.054 (2)	0.057 (2)	0.0035 (17)	0.0024 (19)	−0.0148 (19)
C11	0.0399 (19)	0.061 (2)	0.071 (3)	−0.0074 (17)	0.0068 (19)	−0.013 (2)
C14	0.044 (2)	0.065 (3)	0.067 (2)	0.0038 (18)	−0.006 (2)	−0.011 (2)
O1	0.0590 (17)	0.092 (2)	0.0540 (15)	−0.0015 (16)	−0.0059 (17)	0.0190 (16)
C5	0.049 (2)	0.070 (3)	0.070 (3)	0.007 (2)	−0.011 (2)	0.002 (2)
C4	0.047 (2)	0.064 (2)	0.079 (3)	−0.0066 (19)	0.004 (2)	−0.008 (2)
C13	0.047 (2)	0.060 (2)	0.056 (2)	−0.0005 (17)	0.0015 (18)	0.007 (2)
N2	0.066 (2)	0.097 (3)	0.105 (3)	0.012 (2)	−0.032 (2)	−0.004 (2)

### Geometric parameters (Å, °)

**Table:**

N1—C7	1.277 (4)	C3—H3A	0.9300
N1—C8	1.418 (4)	C2—O1	1.351 (4)
C1—C2	1.396 (4)	C12—C11	1.372 (5)
C1—C6	1.398 (5)	C12—C13	1.382 (5)
C1—C7	1.455 (4)	C12—H12A	0.9300
C9—C8	1.390 (4)	C10—C11	1.381 (5)
C9—C10	1.391 (4)	C10—C14	1.439 (5)
C9—H9A	0.9300	C11—H11A	0.9300
C6—C5	1.375 (5)	C14—N2	1.137 (5)
C6—H6A	0.9300	O1—H1B	0.82 (2)
C8—C13	1.377 (5)	C5—C4	1.374 (5)
C7—H7A	0.9300	C5—H5A	0.9300
C3—C4	1.376 (5)	C4—H4A	0.9300
C3—C2	1.378 (4)	C13—H13A	0.9300
			
C7—N1—C8	120.8 (3)	C3—C2—C1	119.5 (3)
C2—C1—C6	119.0 (3)	C11—C12—C13	120.2 (3)
C2—C1—C7	122.2 (3)	C11—C12—H12A	119.9
C6—C1—C7	118.8 (3)	C13—C12—H12A	119.9
C8—C9—C10	120.5 (3)	C11—C10—C9	119.8 (3)
C8—C9—H9A	119.7	C11—C10—C14	120.6 (3)
C10—C9—H9A	119.7	C9—C10—C14	119.6 (4)
C5—C6—C1	120.9 (4)	C12—C11—C10	119.9 (3)
C5—C6—H6A	119.6	C12—C11—H11A	120.1
C1—C6—H6A	119.6	C10—C11—H11A	120.1
C13—C8—C9	118.6 (3)	N2—C14—C10	178.2 (5)
C13—C8—N1	125.8 (3)	C2—O1—H1B	108 (5)
C9—C8—N1	115.6 (3)	C4—C5—C6	119.3 (4)
N1—C7—C1	121.9 (3)	C4—C5—H5A	120.4
N1—C7—H7A	119.0	C6—C5—H5A	120.4
C1—C7—H7A	119.0	C3—C4—C5	120.8 (3)
C4—C3—C2	120.5 (4)	C3—C4—H4A	119.6
C4—C3—H3A	119.7	C5—C4—H4A	119.6
C2—C3—H3A	119.7	C12—C13—C8	121.0 (3)
O1—C2—C3	119.1 (3)	C12—C13—H13A	119.5
O1—C2—C1	121.4 (3)	C8—C13—H13A	119.5

### Hydrogen-bond geometry (Å, °)

**Table:**

*D*—H···*A*	*D*—H	H···*A*	*D*···*A*	*D*—H···*A*
O1—H1B···N1	0.82 (2)	1.89 (4)	2.623 (4)	149 (7)
